# *USP8*,* USP48*,* BRAF* and *TP53* mutations in crooke cell adenoma

**DOI:** 10.1007/s11102-025-01566-5

**Published:** 2025-10-05

**Authors:** Paulina Kober, Magdalena Szczepaniak, Monika Pękul, Natalia Rusetska, Beata J. Mossakowska, Artur Kowalik, Maria Maksymowicz, Grzegorz Zieliński, Jacek Kunicki, Mateusz Bujko

**Affiliations:** 1 Department of Molecular and Translational Oncology, Maria Sklodowska-Curie National Research Institute of Oncology, Warsaw, Poland; 2 Department of Molecular Diagnostics, Holy Cross Cancer Center, Kielce, Poland; 3 Department of Cancer Pathomorphology, Maria Sklodowska-Curie National Research Institute of Oncology, Warsaw, Poland; 4 Department of Experimental Immunotherapy, Maria Sklodowska-Curie National Research Institute of Oncology, Warsaw, Poland; 5 Department of Genetic Engineering, Holy Cross Cancer Center, Kielce, Poland; 6 Division of Medical Biology, Institute of Biology, Jan Kochanowski University, Kielce, Poland; 7 Department of Neurosurgery, Military Institute of Medicine - National Research Institute, Warsaw, Poland; 8 Department of Neurosurgery, Maria Sklodowska-Curie National Research Institute of Oncology, Warsaw, Poland

**Keywords:** Corticotroph PitNET, Crooke cell adenoma, *USP8*, *USP48*, *BRAF*, *TP53*

## Abstract

**Purpose:**

Crooke cell adenomas (CCAs) are rare histological subtype of corticotroph pituitary adenomas (cPAs) commonly related to worse prognosis in patients. Notable progress in understanding of the molecular background of cPAs has been made recently but biology of CCAs remains poorly recognized. Results of our previous study suggested distinct frequency of the known recurrent mutations in CCAs than in sparsely and densely granulated cPAs. Thus, the aim was to determine the prevalence of *USP8*, *USP48*, *BRAF* and *TP53* variants in a relatively large retrospective group of patients diagnosed with CCA.

**Methods:**

DNA was isolated from formalin-fixed and paraffin-embedded tissue of 29 CCAs (14 clinically functioning and 15 nonfunctioning). Sanger sequencing was used for the identification of *USP8*, *USP48*, *BRAF* hotspot variants, while semiconductor sequencing with Ion AmpliSeq TP53 Panel was used for analysis of *TP53* sequence.

**Results:**

*USP8* variants were found in 2 CCA patients with Cushing’s disease (CD), whereas 3 *TP53* variants were identified in 1 CCA patient with CD and 2 patients with clinically nonfunctioning CCAs. *USP8* variants are less frequent in clinically functioning CCAs than functioning sparsely and densely granulated corticotroph tumors (*p* = 0.0271). *TP53* variants are more common in CCAs as compared to other histological subtypes (*p* = 0.0164). One *BRAF* V600E variant and no *USP48* variant were found.

**Conclusion:**

CCAs have slightly distinct mutational profile then other histological subtypes of cPAs. Since clinical relevance of *TP53* variants in corticotroph tumors was already documented, testing toward *TP53* sequence changes in patients with CCAs should be considered.

**Supplementary Information:**

The online version contains supplementary material available at 10.1007/s11102-025-01566-5.

## Introduction

Pituitary adenomas (PAs) arise from different secretory cells of pituitary gland including corticotroph ACTH-secreting cells. According to current WHO classification 3 histological subtypes of corticotroph PAs are distinguished: sparsely and densely granulated as well as Crooke cell adenomas (CCAs) [1]. CCAs are rare subtype commonly related to worse prognosis [1, 2]. They can be clinically manifested with Cushing’s disease (CD) or with neurological deficits caused by silent corticotroph adenoma (SCA) [3, 4]. Low prevalence of these tumors results in poor understanding of their biological background.

In the last few years a genetic profile of corticotroph PAs has been established as the studies showed recurrent variants in *USP8* [5–7] as well as some other, much less frequent variants in *USP48* [8, 9], *BRAF* [9] and *TP53* [8] that were found in *USP8*-wild type tumors. *USP8* variants appear to determine tumor molecular profile [10, 11] and response to particular pharmacological treatment [12–14], while *TP53* variants were found prognostically relevant [15]. We recently screened a series of 147 corticotroph tumors for variants in protein deubiquitinase-encoding genes, *BRAF* and *TP53*. We included 5 CCAs and interestingly 2 of them were *TP53*-mutated that suggested CCAs being enriched for *TP53* variants [16]. This result prompted us to expand this mutational screening in possibly large retrospective group of CCAs.

## Patients and methods

**Fig. 1:**
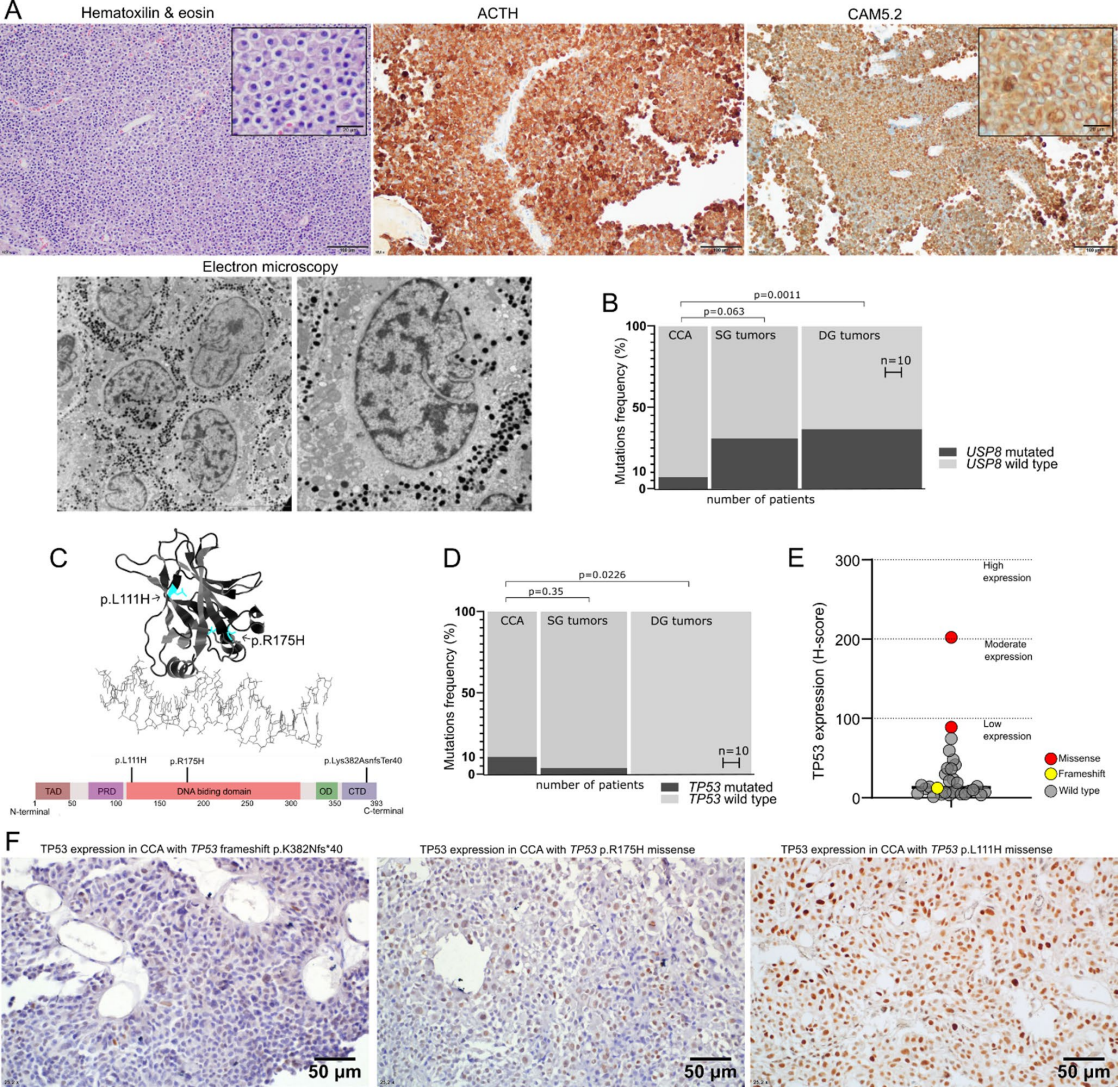
Results of mutational analysis and the assessment of p53 expression in Crooke cell tumors. (**A**) The example of corticotroph Crooke cell adenoma (CCA) including hematoxylin-eosin staining, immunohistochemical staining against ACTH and low molecular weight keratin (Cam5.2 antibody) as well as electron microscopy image of cytokeratin fibrils accumulated around the cell nucleus; magnification x200 (light microscope), magnification x4200 and x9700 (electron microscope). (**B**) The proportion of *USP8*-mutated and *USP8*-wild type CCAs, sparsely granulated (SG) and densely granulated (DG) corticotroph adenomas (including both functioning and silent tumors); (**C**) The location of *TP53* variants identified in Crooke cell adenomas visualized in monomeric p53 structure and map of functional domains. TAD - transactivation domain; PRD - proline-rich domain; OD – oligomerization domain; CTD - carboxy-terminal domain; (**D**) Comparison of the proportion of TP53 mutated tumors in Crooke cell adenomas (CCA), sparsely granulated (SG) and densely granulated (DG) corticotroph tumors (including both functioning and silent tumors); (**E**) p53 expression in Crooke cell adenomas, as assessed with immunohistochemistry and H-score calculation. Mutated *TP53* tumors were marked with colored points; (**F**) The expression of p53 in CCAs with *TP53* variants

The study included 29 patients with tumors that met histological criteria of CCA [1] (as exampled in Fig. 1A). All the tumors were ACTH- and TPIT-positive confirming corticotroph-lineage origin. Crooke’s hyalinization was assessed with both electron microscopy and immunostaining with CAM5.2 antibody.

CD was diagnosed according to previously described criteria [10] in 14 patients. Fifteen patients had SCAs without signs or symptoms of hypercortisolemia, no history of exogenous glucocorticosteroid treatment and were qualified for pituitary surgery due to symptoms of pituitary tumor mass growth. Five patients were already included in our previous study group [16].

Variants in *USP8*,* USP48* and *BRAF* were analyzed with Sanger sequencing while [16] *TP53* sequence was analyzed with targeted next-generation sequencing using predesigned Ion AmpliSeq TP53 Panel (Thermo Fisher Scientific). These methods were used in our previous study [16]. Immunohistochemistry with Envision Detection System (DAKO) and anti-human p53 antibody (clone DO-7) was applied to visualize p53 expression. Nuclear immunoreactivity was quantified by calculating H-score (ranged between 0 and 300). Three high power fields were evaluated. Description of the study group and methodology is presented in Supplementary File 1.

## Results

We found only two *USP8* variants (p.S718SP, p.P720R) in female patients with CCA, both with clinical features of CD (27 and 34 years old, respectively). Both tumors were noninvasive (Knosp’s grade 2 and 1) microadenomas. We compared frequency of *USP8* variants in CCAs with sparsely granulated (SG) (*n* = 46) and densely granulated (DG) (*n* = 93) corticotroph PAs that were analyzed previously [16]. *USP8* variants were found in 6.9% (2/29) of CCAs, 23,9% (11/46) of SG PAs and 36.55% (34/93) of DG PAs (Fig. 1B), with significant difference between CCAs and DG PAs (Fisher’s exact test *p* = 0.0011) but not between CCAs and SG tumors (*p* = 0.063). Because *USP8* mutations are rare in SCAs [10] we also compared their frequency in functioning tumors only. *USP8* variant occurred in 14.3% (2/14) of functioning CCAs, 31% (9/29) of functioning SG and 48.5% (32/66) of functioning DG adenomas with significant difference between CCAs and DG tumors (*p* = 0.0344). No *USP48* variant was found. One *BRAF* p.V600E variant was identified in 65 year-old woman, with recurrent nonfunctioning, macroadenoma.

*TP53* sequence was analyzed in all but one patient, excluded due to low sequence readout quality. Three variants have been identified, each in one patient: two were missense variants p.L111P and p.R175H (both localized in TP53 DNA-binding domain) and frameshift p.K382Nfs*40 (located in carboxy-terminal domain) (Fig. 1C). Functional classification of the variants is presented in Table 1. Additionally, in one patient we identified P85S variant of uncertain significance, localized in proline-rich domain, disordered region with unclear functional relevance [17]. It was not considered as “pathogenic”/”possibly pathogenic” due to poor functional evidence and low variant allelic frequency of 3%.

**Table 1:** Functional classification of *TP53* variants identified in Crooke cell adenomas according to public databases on the relationships between human genetic variations and their clinical significance

Variant	Franklin ACMG	Franklin somatic	Varsome	Clinvar	UMD TP53
p.L111P	Pathogenic	Tier 1	Pathogenic	Conflicting classifications of pathogenicity Likely pathogenic(3); Uncertain significance(1)	Possibly pathogenic
p.R175H	Pathogenic	Tier 1	Pathogenic	Pathogenic	Pathogenic
p.K382Nfs*40	Likely Pathogenic	Tier 1	Pathogenic	Uncertain significance	Likely Pathogenic
p.P85S	VUS	Tier 2	Likely Benign	Uncertain significance	VUS

One *TP53*-mutated CCA was clinically functioning while two remaining mutated tumors were nonfunctioning. All *TP53* variants were found in invasive (Knosp grade 4 in one and grade 3 in two patients) macroadenomas. In medical history there was no information suggesting a possible genetic burden predisposing to early cancer onset due to germline *TP53* variant in these patients. *TP53* variants were significantly more common in CCAs that other histological subtypes of corticotroph PAs in previously analyzed group of patients (Fig. 1D). The following frequency of *TP53* variants was observed: 10.3% (3/29) in CCAs, 4.3% (2/46) in SG and 0% (0/93) DG PAs, with significant difference between CCAs and DG corticotroph adenomas (*p* = 0.0124).

Median H-score of p53 immunoreactivity was 12.76 (range 1.2 min. – 202 max.) and the values for all but one sample were below 100, categorized as low expression. Two samples with missense *TP53* variants were rated 88.9 and 202.1 (low and high expression, respectively) while tumor with frameshift was scored 12 (Fig. 1E, F).

## Discussion

Our results show distinct mutational profile of CCAs as compared to SG and DG corticotroph PAs with lower prevalence of *USP8* variants (as reported previously [13]) and higher frequency of *TP53* variants. *USP8*-mutated tumors are molecular subtype of corticotroph PAs with distinct genes expression [10, 11] including slightly different *GR* level [18]. Possibly their distinct biology may influence glucocorticoid excess response and Crooke cell hyalinization.

Changes in *TP53* sequence relate to worse prognosis in corticotroph PAs [15, 16] and occur in aggressive corticotroph PAs and carcinomas [19–25] *TP53* changes. However, the results show they are still infrequent. The literature data on CCAs genetics is very limited and *TP53* sequence was screened only in a few cases so far. Two patients with CCAs were included in the group of 22 aggressive corticotroph PAs that was analyzed with whole exome sequencing by Uzilov et al. [21]. Both tumors were *TP53*-wild type. In turn, *TP53* variant of unknown significance was found in 1 CCA by Andonegui-Elguera et al. [26]. The 10% prevalence of *TP53* variants in CCAs and the fact that the variants are related to worse outcome suggest that screening of patients with CCAs for genetic changes in this gene could be reasonable and may provide important prognostic information. Unfortunately, at the moment we were unable to complete the follow-up information on the patients that could allow for a reliable analysis of clinical relevance of *TP53* variants in our study group. We also believe that a larger patients’ cohort is required. We recognize both above as the main limitations of this report.

For years, positive immunoreactivity against p53 was considered equivalent to mutational screening [27]. Before introducing 4th edition of WHO classification, positive p53 staining was a criterion of atypical pituitary adenoma but some studies failed to confirm its prognostic value [27–29]. Importantly, 3/6 *TP53* variants previously found in *USP8*-wild type CD patients were nonsense variants and deletions that result in protein loss, rather than protein accumulation [8]. In our study only 1/3 *TP53*-mutated tumors had increased protein expression which confirms the need of DNA sequence analysis instead of immunohistochemistry for future *TP53* screening.

## Supplementary Information

Below is the link to the electronic supplementary material.

ESM 1 (PDF 213 KB)

## Data Availability

The data that support the findings of this study are available on request from the corresponding author.
