# Supplementary material for: USP8, USP48, BRAF and TP53 mutations in crooke cell adenoma
Source: Pituitary. 2025 Oct 5;28(5):107. doi: 10.1007/s11102-025-01566-5 (PMC12497664; doi:10.1007/s11102-025-01566-5)
Supplement: Supplementary file 1 — (PDF 213 KB) [file 11102_2025_1566_MOESM1_ESM.pdf]

Supplementary methods

USP8, USP48, BRAF and *TP53* sequence variants in Crooke cell adenoma.

Patients.

The study included 29 adult patients with Crooke cell adenomas including 14 patients with Cushing disease and 15 patients with silent corticotroph adenomas (SCAs). Inclusion criteria was histological diagnosis of corticotroph pituitary adenoma with Crooke cell hyalinization present in more than 50% of the cells as assessed with electron microscopy and confirmed with immunohistochemical staining. All the tumors were ACTH- and TPIT-positive confirming corticotroph-lineage origin. Crooke's hyalinization was confirmed with both electron microscopy and immunostaining with CAM5.2 antibody.

Histological examination for all the patients was performed at Cancer Pathomorphology Department at , National Research Institute of Oncology in Warsaw including immunohistochemical staining and electron microscopy. Immunohistochemical staining against pituitary hormones (ACTH, GH, PRL, TSH, FSH, LH, α-subunit) was performed at the moment of diagnosis for all the patients. Staining against TPIT transcription factor and low molecular weight keratin (Cam5.2 antibody) was complemented for study purpose.

For electron microscopy, tissue samples were fixed in 2.5% glutaraldehyde, postfixed in 1% osmium tetroxide, dehydrated and embedded in epoxy resin (Epon 812). Ultrathin sections counterstained with uranyl acetate and lead citrate were examined with a Philips CM120 BioTWIN transmission electron microscope.

All patients were treated with surgery at the Department of Neurosurgery, National Research Institute of Oncology in Warsaw or Department of Neurosurgery, Military Institute of Medicine - National Research Institute in Warsaw, between years 2004-2020. CD patients had evident clinical signs and symptoms of hypercortisolism verified according to biochemical criteria: increased urinary free cortisol (UFC) in three 24h urine collections; disturbed cortisol circadian rhythm (with midnight serum cortisol level or late night salivary cortisol) or no suppression of serum cortisol levels to <1.8 µg/dL after an overnight dexamethasone suppression test (1 mg at midnight).

The pituitary etiology of Cushing`s syndrome was confirmed based on the measurement of morning serum ACTH level (and additionally by positive result of a corticotropin-releasing hormone stimulation test in patients with normal ACTH level (100 mg i.v.) as well as magnetic resonance imaging of pituitary. In patients with inconclusive MRI bilateral inferior petrosal sinus sampling (BIPSS) was applied.

Patients with SCAs were diagnosed as clinically nonfunctioning pituitary adenomas. The endocrinological work up showed neither signs nor symptoms of hypercortisolemia, no history of exogenous glucocorticosteroid treatment. The hypercortisolemia was excluded and normal cortisol circadian rhythm was assessed with one or two screening tests (increased UFC in three 24h urine collections, no suppression in the late night salivary cortisol or no suppression of serum cortisol levels after an overnight 1 mg dexamethasone suppression test). They all had MRI-confirmed pituitary tumor and were qualified for pituitary surgery due to symptoms of mass effect, visual disturbances or neurological deficits.

ACTH levels were assessed using IRMA (ELSA-ACTH, CIS Bio International, Gif-sur-Yvette Cedex, France) with analytical sensitivity of 2 pg/ml (reference range: 10–60 pg/ml). Serum cortisol concentrations were determined by the Elecsys 2010 electrochemiluminescence immunoassay (Roche Diagnostics, Mannheim, Germany) with sensitivity of the assay of 0.02 µg/dl (reference range: 6.2–19.4 µg/dl). UFC was determined after extraction (liquid/liquid with dichloromethane) by electrochemiluminescence immunoassay (Elecsys 2010, Roche Diagnostics, Mannheim, Germany) - reference range: 4.3-176 µg/24h.All the functioning tumors and SCA samples were ACTH-positive in immunohistochemical staining against pituitary hormones (ACTH, GH, PRL, TSH, FSH, LH, α-subunit) and had characteristic ultrastructural features of corticotroph tumors as determined with electron microscopy. SCA samples were subjected to additional immunostaining against TPIT to verify the corticotroph origin of the tumors.

Five CCAs included in these study was already described in our previous article [1]. These five CCAs included two cases with TP53 mutations. To compare CCAs with sparsely and densely granulated corticotroph adenomas

we used data published in the previous article.[1]. Data from 46 sparsely granulated corticotroph adenomas (including 29 clinically functioning adenomas and 17 SCAs) and 93 densely granulated corticotroph adenomas (including 66 clinically functioning adenomas and 27 SCAs) was used.

Invasive tumor growth status was assessed based on MR imaging using Knosp's scale [2]. Tumors with Knosp grades 0,1 and 2 were considered non-invasive, whereas tumors with Knosp grades 3 and 4 were considered invasive.

Supplementary Table 1. Patients characteristics

**Clinical Feature**	**Cushing's disease**	**Silent corticotroph adenoma (SCA)**
**Number of patients**	n=14	n=15
**Sex**<br>females<br>males	 12<br>2	 5<br>8
**Age at surgery** (years; median (range))	55 (18-67)	55 (19-77)
**Cortisol 08:00 h** (µg/dL; median (range); reference range :6.2-19.4 µg/dL)	26.7 (17.9 – 34.24)	13.5 (4 – 18.6)
**ACTH 08:00 h** (pg/dL; median (range); (reference range: 10-60 pg/mL)	69.5 (42.9 – 305)	29.1 (4.2 – 104) *
**UFC** (µg/24 h; median (range); (reference range: 4.3-176 µg/24h)	371 (160 – 738)	113 (91 - 164)**
**Tumor largest size** (mm; median (range))	15 (6 - 36)	24 (14 – 53)
**Invasive tumor growth**<br>Non-invasive (Knosp grade 0, 1, 2)<br>invasive (Knosp grade 3, 4)	 8<br>6	 8<br>7
**primary/recurrent tumor**<br>Newly diagnosed<br>Recurrent	 12<br>2	 14<br>1
**Surgery extent**<br>Gross total surgery<br>Subtotal surgery<br>Partial surgery	 9<br>4<br>1	 11<br>4<br>-

*not available for 3 patients with SCA; **not available for 9 patients with SCA

DNA isolation

Genomic DNA was isolated from formalin-fixed and paraffin-embedded (FFPE) tumor tissue samples using RecoverAll™ Total Nucleic Acid Isolation Kit for FFPE [Thermo Fisher Scientific, Waltham, Massachusetts, USA], measured using NanoDrop 2000 [Thermo Fisher Scientific, Waltham, Massachusetts, USA] and stored at -70°C.

Sanger sequencing

Sequence of *USP8* (coding for 14-3-3 binding motif, chr15:50,490,362-50,490,596; hg38), *USP48* (chr1:21,729,673-21,729,899; hg38) and *BRAF* (chr7:140,753,278-140,753,450; hg38) were analyzed with Sanger sequencing. DNA was PCR-amplified with FastStart Taq DNA Polymerase (Roche Diagnostics, Mannheim, Germany) using GeneAmp 9700 PCR system (Applied Biosystems, Foster City, CA, USA). PCR product was purified using ExoStar (GE Healthcare Life Sciences, Pittsburgh, PA, USA), labeled with BigDye Terminator v.3.1 (Applied Biosytems, Foster City, CA, USA) according to the manufacturer's instructions and analyzed by capillary electrophoresis with the ABI PRISM 3300 Genetic Analyzer (Applied Biosystems, Foster City, CA, USA). The following nucleotide/protein sequences were used as reference: NM_005154.5/NP_005145.3; NM_032236.8/NP_115612.4 and NM_004333.6/NP_004324 for detecting variant in USP8, USP48 and BRAF, respectively.

TP53 sequence analysis with targeted next generation sequencing (NGS)

Supplementary methods

The TP53 gene was sequenced by NGS using Ion Torrent technology. For this purpose, a pre-designed panel Ion AmpliSeq TP53 Panel (TP53.20140108, Ion AmpliSeq Community Panel) covering the entire protein-coding sequence of TP53 and exon-intron boundaries was selected by the online tool Ion Ampliseq Designer (Thermo Fisher Scientific).

Sample concentrations were measured using Qubit 2.0 Fluorometer (Life Technologies) with the dsDNA HS Assay Kit. Next, according to the manufacturer's instructions, gene libraries were prepared using Ion AmpliSeq™ Library Kit Plus (Thermo Fisher Scientific) and the primers. Sequencing was performed using Ion GeneStudio™ S5 Prime System (Thermo Fisher Scientific).

The designed panel detects single nucleotide polymorphisms, as well as deletions and insertions of up to 20 nucleotides. The method's limit of detection is ≥ 5% at the 99% confidence level. Detection of homopolymeric variants is also limited due to specificity of Ion Torrent technology.

To analyze the results generated, free tools available online wAnnovar (https://wannovar.wglab.org/) and Sheshat (https://p53.fr/tp53-database/seshat) were used to describe the obtained variants. For the analysis, Integrative Genomics Viewer (https://igv.org/) was also used to provide a visual representation of the detected alterations. The filtered variants were then checked using the Clinvar (https://www.ncbi.nlm.nih.gov/clinvar/) and the UMD TP53 (https://p53.fr/download-the-database) dedicated to the TP53 gene database, as well as the Varsome bioinformatics tool (https://varsome.com/). Classification of mutations was based mainly on the interpretation of their significance provided by Clinvar and UMD TP53 (https://p53.fr/the-database), and if a particular alteration was not classified, the Varsome tool was used to determine pathogenicity according to ACMG guidelines. Nucleotide/protein sequences NM_000546.6/ NP_000537.3 were used as reference.

Immunohistochemistry

Immunohistochemical staining (IHC) was applied to visualize p53 expression level in 29 patients. Envision Detection System (DAKO, Glostrup, Denmark) was used for staining procedure according to manufacturer's recommendations. Four-µm FFPE tissue sections were deparaffinized with xylene and rehydrated in a series of ethanol solutions of decreasing concentration. Heat-induced epitope retrieval was achieved by 30 minutes incubations of the samples in Target Retrieval Solution pH 9 (DAKO) in a 96°C water bath. The slides were treated with a Blocker of Endogenous Peroxidase (DAKO) for 5 minutes followed by incubation with the primary monoclonal mouse anti-human TP53 antibody (clone DO-7, ready to use concentration, DAKO) for 1h. Diaminobenzidine tetrahydrochloride (Dako) was used as substrate to visualize immunoreactivity followed by hematoxylin nuclear counterstaining. Analysis of nuclear immunohistochemical reactivity was performed by calculating H-score, that combines information on both reaction intensity (scored from 0 to 3) and number of the cells with a given intensity. The following formula was used:

H-score=(1×% of cells with intensity 1)+(2×% of cells with intensity 2)+(3×% of cells with intensity 3)

Three high power fields (magnification ×400) were evaluated for each sample. Scoring results were analyzed as continuous variables and calorized as no expression (H-score=0), low expression (H-score range: 1-100), moderate expression (H-score range: 101-200) and high expression (H-score range: 201-300).

Statistical analysis

Percent of the patient were used as descriptive statistics. Two sided Fisher's exact test was applied for the analysis of proportions in 2×2 tables. Significance threshold of $\alpha = 0.05$ was adopted. Data was analyzed using GraphPad Prism 6.07 (GraphPad Software).

References

1. Pękul M, Szczepaniak M, Kober P, et al (2024) Relevance of mutations in protein deubiquitinases genes and TP53 in corticotroph pituitary tumors. Front Endocrinol (Lausanne) 15:. https://doi.org/10.3389/fendo.2024.1302667

Supplementary methods

2. Knosp E, Steiner E, Kitz K, et al (1993) Pituitary adenomas with invasion of the cavernous sinus space: A magnetic resonance imaging classification compared with surgical findings. Neurosurgery 33:610–618. https://doi.org/10.1017/CBO9781107415324.004
